# Analysis of a circRNA-, miRNA-, and mRNA-associated ceRNA network reveals potential biomarkers in preeclampsia a ceRNA network in preeclampsia

**DOI:** 10.1080/07853890.2021.2014554

**Published:** 2021-12-13

**Authors:** Xiaoxiao Xu, Sha Lv, Ziwen Xiao

**Affiliations:** Guizhou Medical University, Guiyang City, China

**Keywords:** Preeclampsia, ceRNA, circRNA, miRNA, bioinformatics analysis

## Abstract

**Background:**

Preeclampsia (PE), one of hypertension-related disorders of pregnancy, is a common cause of maternal death worldwide. This study aimed to identify a circRNA-miRNA-mRNA-associated ceRNA network and related pathways in PE.

**Material and methods:**

We downloaded 3 microarray datasets from the Gene Expression Omnibus database, obtained 163 differentially expressed circRNAs (dif-circRNAs) (61 upregulated and 102 downregulated), 39 differentially expressed microRNAs (dif-miRNAs) (22 upregulated and 17 downregulated), and 271 differentially expressed mRNAs (dif-mRNAs) (168 upregulated and 103 downregulated) from placenta tissues of PE. Functional enrichment analysis and protein-protein interaction (PPI) network with module analysis of dif-mRNAs were performed. The regulatory relationship between dif-miRNAs and dif-mRNAs/circRNAs was predicted *via* related databases. A circRNA-miRNA-mRNA regulatory network was constructed.

**Results:**

A total of 53 pairs were obtained, including 13 circRNAs (10 upregulated and 3 downregulated), 9 miRNAs (3 upregulated and 6 downregulated) and 31 mRNAs (22 upregulated and 9 downregulated). GNB5 and IL2RB were obtained. KEGG enrichment analysis showed that both of them were closely related with the PI3K-Akt signalling pathway. Therefore, ceRNAs might affect the PI3K-Akt signalling pathway *via* the upregulation of GNB5 by binding to miR-1248 in PE. Meanwhile, hsa_circ_0052661 might upregulate IL2RB by binding miR-4303 to play a role in PE in the same way.

**Conclusion:**

GNB5 and IL2RB might be key genes involved in the PI3K-Akt signalling pathway in PE, and hsa_circ_0087208, hsa_circ_0035443, hsa_circ_0067557 and hsa_circ_0052661 might regulate these key genes in PE by binding miR-1248 or miR-4303.

**Key messages**
- There is still a lack of predictive and diagnostic factors for preeclampsia, which is a common cause of maternal death worldwide.
- This study identified a novel circRNA-associated ceRNA network and related pathways in preeclampsia.
- GNB5 and IL2RB might be key genes in their related circRNA-associated ceRNA network, and probably take an important role in preeclampsia via PI3K-Akt signalling pathway, which made them to be potential markers of preeclampsia.

## Background

Preeclampsia (PE) has an increasing incidence worldwide and remains a high cause of morbidity and mortality in pregnant women and foetuses in developing countries [1]. PE may cause serious complications, such as eclampsia, cerebral haemorrhage, renal failure, disseminated intravascular coagulation (DIC), pulmonary edoema, and even maternal and foetal death [2]. PE manifests as hypertension and proteinuria after 20 weeks of pregnancy [3], which occur only during pregnancy. Once symptoms appear, the disease has already taken a certain toll on the pregnant woman and the foetus. Therefore, the prediction and early diagnosis of the disease are crucial. Although there are numerous basic and clinical studies about PE, its aetiology remains obscure. And due to its elusive pathophysiology, PE still lacks a suitable biomarker for diagnosis and prediction.

It has been reported that some genes and noncoding RNAs, including microRNA (miRNA), long noncoding RNA (lncRNA) and circular RNA (circRNA), are related to the occurrence and development of PE [4–6]. MiRNAs have been reported to be important for the regulation of cellular processes by downregulating their target gene expression in PE, which also made them had the potential clinical utility as non-invasive biomarker [7]. Meanwhile, CircRNAs have emerged as important factors in PE, for their contributing to regulate gene transcription and mRNA translation, etc. Competing endogenous RNA (ceRNA) network is one of the most important mechanisms *via* which the miRNAs and circRNAs take part in gene regulation. CircRNAs may serve as miRNA sponges to liberate mRNAs regulated by the miRNAs [8]. However, very limited research has been performed on ceRNA networks composed of circRNAs, miRNAs, and mRNAs. Therefore, we explored circRNAs to identify new candidate biomarkers as targets for prediction and early diagnosis of PE.

This study aimed to identify a circRNA-miRNA- mRNA-associated ceRNA network and related pathways in PE. In order to do that, we analyzed 3 datasets from a public database to screen for the differentially expressed circRNAs (dif-circRNAs), differentially expressed miRNAs (dif-miRNAs) and differentially expressed mRNAs (dif-mRNAs) in placental tissues of the PE and control groups. PPI network analysis and an analysis of the enrichment of the functions and signalling pathways of the differentially expressed genes were performed. We explored the regulatory network analysis of miRNA with mRNA/circRNA to construct and analyze the circRNA-miRNA-mRNA in PE.

## Material and methods

### Data sources

The public datasets GSE96984 (this study has not yet been published), GSE103542 [9] and GSE147776 [10] were downloaded from the Gene Expression Omnibus (GEO) database (https://www.ncbi.nlm.nih.gov/geo/) [11]. All datasets contained placental tissues of PE patients and the control group. More details are available in Table 1.

**Table 1.** Details of three datasets downloaded from GEO database.

	GSE96984	GSE103542	GSE147776
Status	Public on 1 March 2018	Public on 7 September 2017	Public on 31 May 2020
Title	Different expression profiles of lncRNAs, mRNAs and circRNAs in preeclampsia and normal placenta (ceRNA)	Dysregulated Placental Micrornas In Early And Late Onset Preeclampsia	Placental microarray profiling reveals common mRNA and lncRNA expression patterns in Preeclampsia and Intrauterine Growth Restriction
Experiment type	Non-coding RNA profiling by array; Expression profiling by array	Non-coding RNA profiling by array	Expression profiling by array
Platforms	GPL22120: Agilent-078298 human ceRNA array V1.0 4X180K [Probe Name Version]	GPL23980: miRLink microRNA Arrays v. 16	GPL20844: Agilent-072363 SurePrint G3 Human GE v3 8x60K Microarray 039494 [Feature Number Version]
Tissue Type	7 placental samples from 3 preeclampsia patients and 4 normal women were enrolled in. Focused on the basal plate of placenta, around the umbilical cord	24 placental samples from 16 PE patients and 8 with uncomplicated term pregnancies From the central part of the placenta	placental samples from: Preeclampsia (n = 7), Preeclampsia and Intrauterine Growth Restriction (n = 6); Normal Pregnancy (n = 8), Intrauterin Growth restriction (n = 7)^a^ Full-thickness placental tissue was taken from the middle region of the placenta, close to the umbilical cord insertion
Contributor(s)	Wang Q, Zhang Z, Liu C	Lykoudi A, Kolialexi A, Lambrou GI, et al.	Medina-Bastidas D, Salido-Guadarrama I

^a^This group was not included in our study.

### Analysis of differential expression

The primary data were preprocessed using Perl [12] and the Limma package in R software to screen the differentially expressed circRNAs, miRNAs and mRNAs between PE and control placental tissues [13]. Gene expression values were calculated by mapping probes to symbols utilizing the microarray dataset and the Supplementary files from the platform. Probe sets with more than one gene symbol or without the corresponding gene symbol were removed. Genes with two or more probe sets were averaged. The cut-off criteria were different for large differences in quantity. For circRNA, the criteria were: adj. *p* value <.01 and log Fold Change (logFC) >3. For miRNA, as the criteria were: *p* value <.05 and logFC >1. In addition, the criteria for mRNA were as follows: adj. *p* value <.05 and logFC >1.

### Protein-protein interaction network and module analyses

The PPI network of dif-mRNAs was preprocessed using the Search Tool for the Retrieval of Interacting Genes (STRING; http://string-db.org) online database (version 11.0) [14] and visualized by Cytoscape 3.8.0 [15]. A statistically significant interaction was observed with a combined score > 0.4. The modules were screened by the Molecular Complex Detection (MCODE) plugin [16] with the following selection criteria: MCODE scores > 5, degree cut-off = 2, node score cut-off = 0.2, max depth = 100 and *k*-score = 2.

### Function and pathway enrichment analysis

Gene Ontology (GO) and Kyoto Encyclopedia of Genes and Genomes (KEGG) enrichment analyses of the dif-mRNAs and mRNAs in the circRNA-miRNA-mRNA network were performed using the clusterProfiler tool in the R package [17]. The GO knowledge base describes the information on the functions of genes in three independent ontologies [18,19]: biological process (BP), molecular function (MF) and cellular component (CC). KEGG database offers resource of genomic, chemical and systemic functional information, which is widely used as a reference knowledge base for integration and interpretation of large-scale datasets generated by genome sequencing and other high-throughput experimental technologies [20]. *p* < .05 was considered statistically significant in the function and pathway enrichment analysis.

### Regulatory relation analysis of miRNA with mRNA/circRNA

The dif-circRNA and dif-miRNA pairs were predicted using circBank (http://www.circbank.cn/), which is a comprehensive database of human circRNAs that includes more than 140,000 human annotated circRNAs from different sources [21]. The target mRNAs for dif-miRNAs were predicted using R based on two miRNA databases, TargetScan (http://www.targetscan.org/mamm_31/) and miRDB (http://mirdb.org/). TargetScan predicts miRNA target genes by searching for the presence of 6- to 8-mer sites that match the seed region of a given miRNA [22]. All the targets in miRDB were predicted by a bioinformatics tool, MirTarget, which was developed by analyzing thousands of miRNA-target interactions from high-throughput sequencing experiments [23]. Only genes that were predicted by both databases were included.

### CircRNA–miRNA–mRNA integration analysis

The complex integrated networks of circRNA–miRNA–mRNAs were constructed by using Cytoscape tools containing the obtained circRNA–mRNA, miRNA–circRNA, and miRNA–mRNA pairs.

## Results

### Differential expression analysis

**Figure 1.**
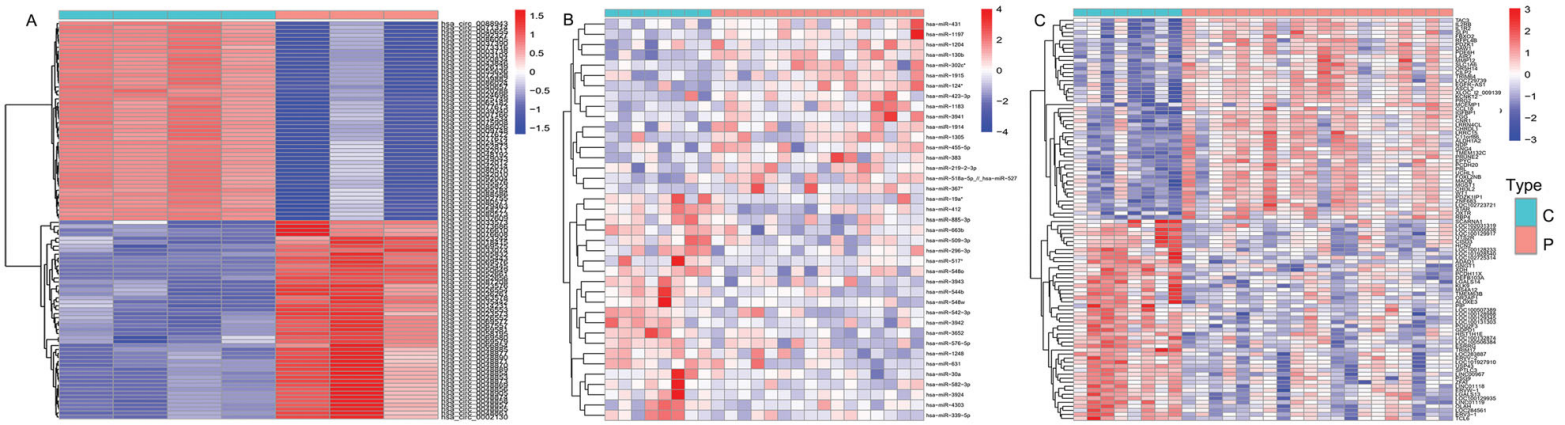
The bidirectional clustering heat maps of dif-circRNAs (A), dif-miRNAs (B), and dif-mRNAs (C). C: control group; P: PE group; Dif- circRNAs/miRNAs/mRNAs are shown in different shades.

The bidirectional clustering heat maps of dif-circRNAs, dif-miRNAs, and dif-mRNAs were drawn by R and are shown in Figure 1. Based on our filter criteria, there were 163 dif-circRNAs (61 upregulated and 102 downregulated), 39 dif-miRNAs (22 upregulated and 17 downregulated), and 271 dif-mRNAs (168 upregulated and 103 downregulated) between PE and control samples.

### PPI network and module analyses of dif-mRNAs

**Figure 2.**
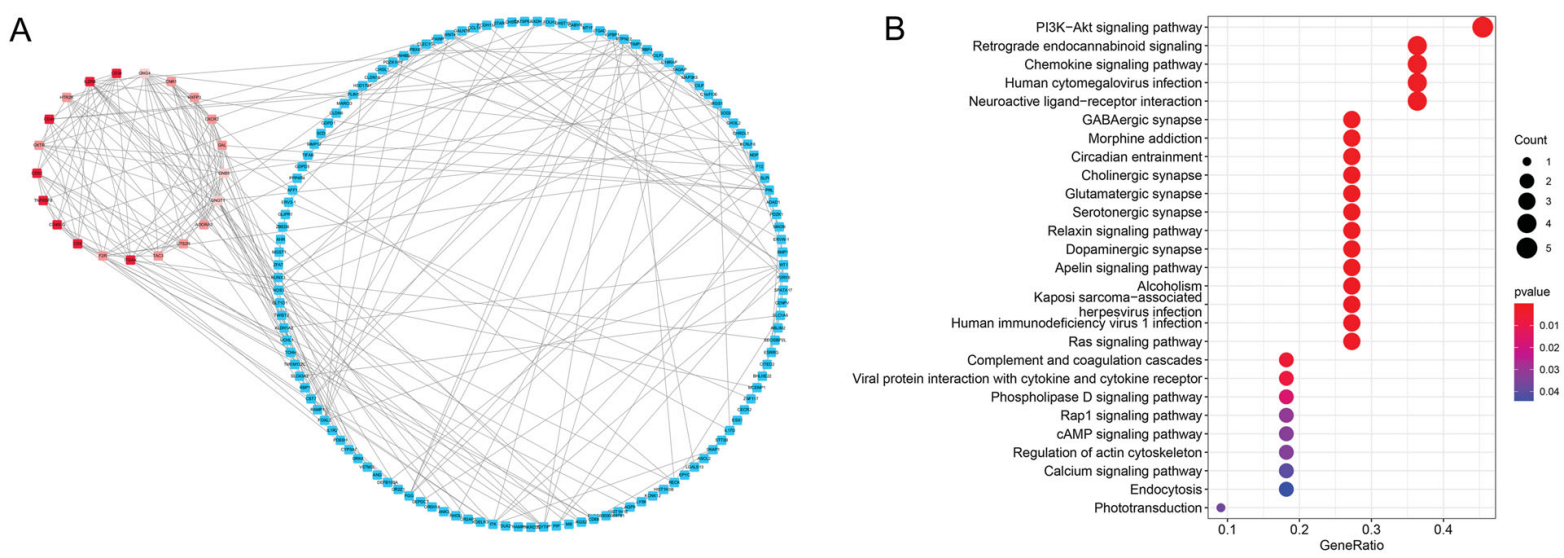
PPI network and KEGG enrichment of dif-mRNAs. (A) PPI network of dif-mRNAs. Three modules are marked in various shades of red. (B) KEGG enrichment of 12 nodes with the degree ≥10 in modules.

The PPI network of dif-mRNAs was constructed using Cytoscape, containing total 146 nodes and 256 edges. Three modules with total 18 nodes was obtained by MCODE (Figure 2(A)), in which 12 nodes of them with a degree of ≥10 (Table 2). KEGG enrichment analysis showed that most of the 12 genes were involved in PI3K-Akt signalling pathway (Figure 2(B)).

**Table 2.** Details of 12 genes with a degree of ≥10 in 3 modules.

Name	Degree	MCODE_Cluster	MCODE_Score	Gene title
GNGT1	19	Cluster 3	4.935897	Guanine nucleotide-binding protein G(T) subunit gamma-T1
GNB5	16	Cluster 3	4.935897	Guanine nucleotide-binding protein subunit beta-5
GNG4	15	Cluster 3	4.935897	Guanine nucleotide-binding protein subunit beta-4
IL2RB	14	Cluster 1	5	Interleukin-2 receptor subunit beta
ITGAX	14	Cluster 1	5	Integrin alpha-X
F2R	11	Cluster 2	7	Proteinase-activated receptor 1
CNR1	11	Cluster 2	7	Cannabinoid receptor 1
CXCR2	11	Cluster 2	7	C-X-C chemokine receptor type 2
CD5	10	Cluster 1	5	T-cell surface glycoprotein CD5
CD52	10	Cluster 1	5	CAMPATH-1 antigen
OXTR	10	Cluster 2	7	Oxytocin receptor
GAL	10	Cluster 2	7	Galanin peptides

### Function and pathway enrichment analysis of dif-mRNAs

**Figure 3.**
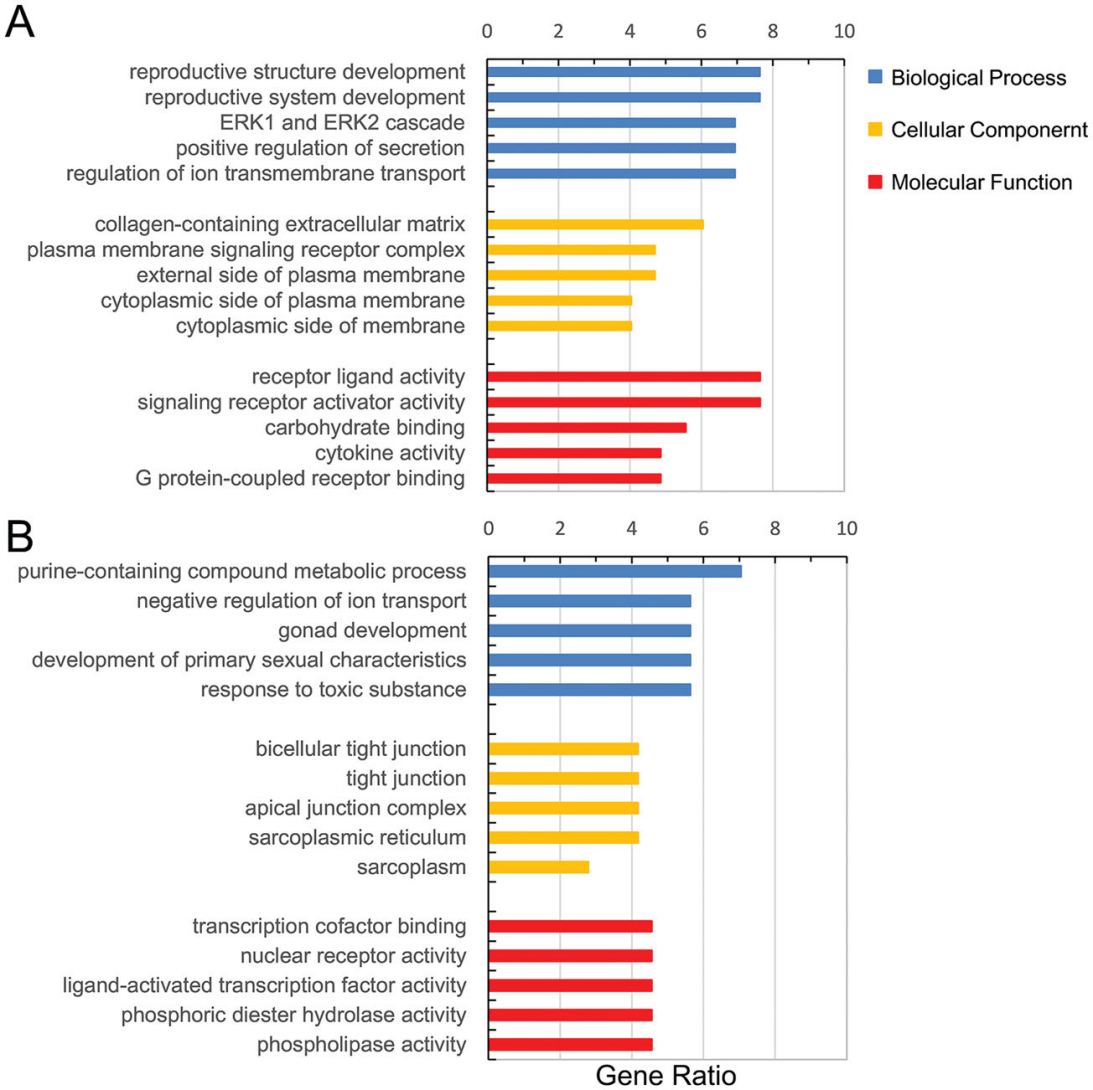
Functional enrichment of dif-mRNAs. (A) Upexpressed dif-mRNAs are mainly associated with reproductive structure development in BP, collagen-containing extracellular matrix in CC and receptor ligand activity in MF. (B) Downexpressed dif-mRNAs are primarily involved in purine-containing compound metabolic process in BP, bicellular tight junction in CC, and transcription cofactor binding in MF.

**Figure 4.**
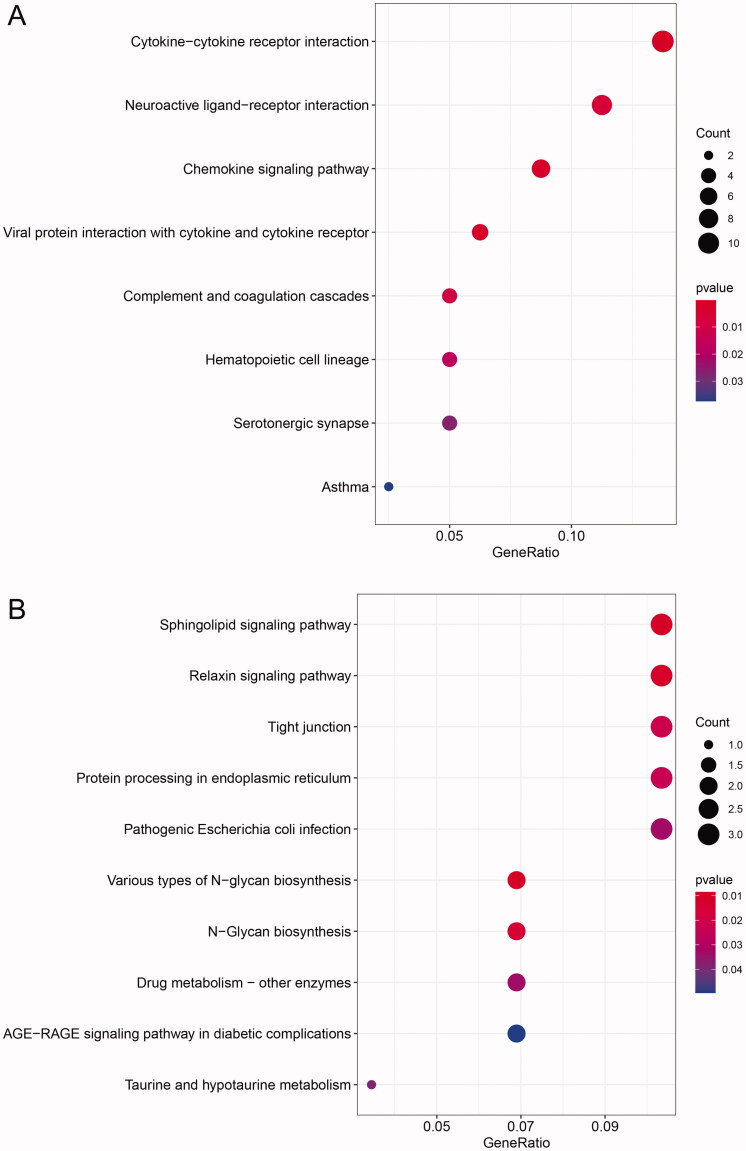
KEGG enrichment analysis of dif-mRNAs. (A) Upexpressed dif-mRNAs were primarily related with cytokine–cytokine receptor interaction pathway. (B) Downexpressed dif-mRNAs were mainly associated with sphingolipid signalling pathway.

GO and KEGG enrichment analyses for the up- and downregulated dif-mRNAs were performed using R. Sorted by gene ratio, the top 5 GO terms of the BP, MF and CC categories are illustrated in Figure 3, separated into the up- and downregulated mRNAs. The 168 upregulated genes were mainly related to the reproductive structure, development reproductive system, and development in the BP category; collagen-containing extracellular matrix in the MF category; and receptor ligand activity and signalling receptor activator activity in the CC category (Figure 3(A)). The 103 downregulated genes were primarily involved in purine-containing compound metabolic process in the BP category; bicellular tight junction, tight junction, and apical junction complex in the MF category; and transcription cofactor binding, nuclear receptor activity, and ligand-activated transcription factor activity in the CC category (Figure 3(B)). KEGG pathway analysis revealed that the upregulated genes were primarily related to the cytokine–cytokine receptor interaction pathway (Figure 4(A)), and the downregulated genes were mainly involved in the sphingolipid signalling pathway (Figure 4(B)).

### Regulatory relation analysis of miRNA with mRNA/circRNA

**Figure 5.**
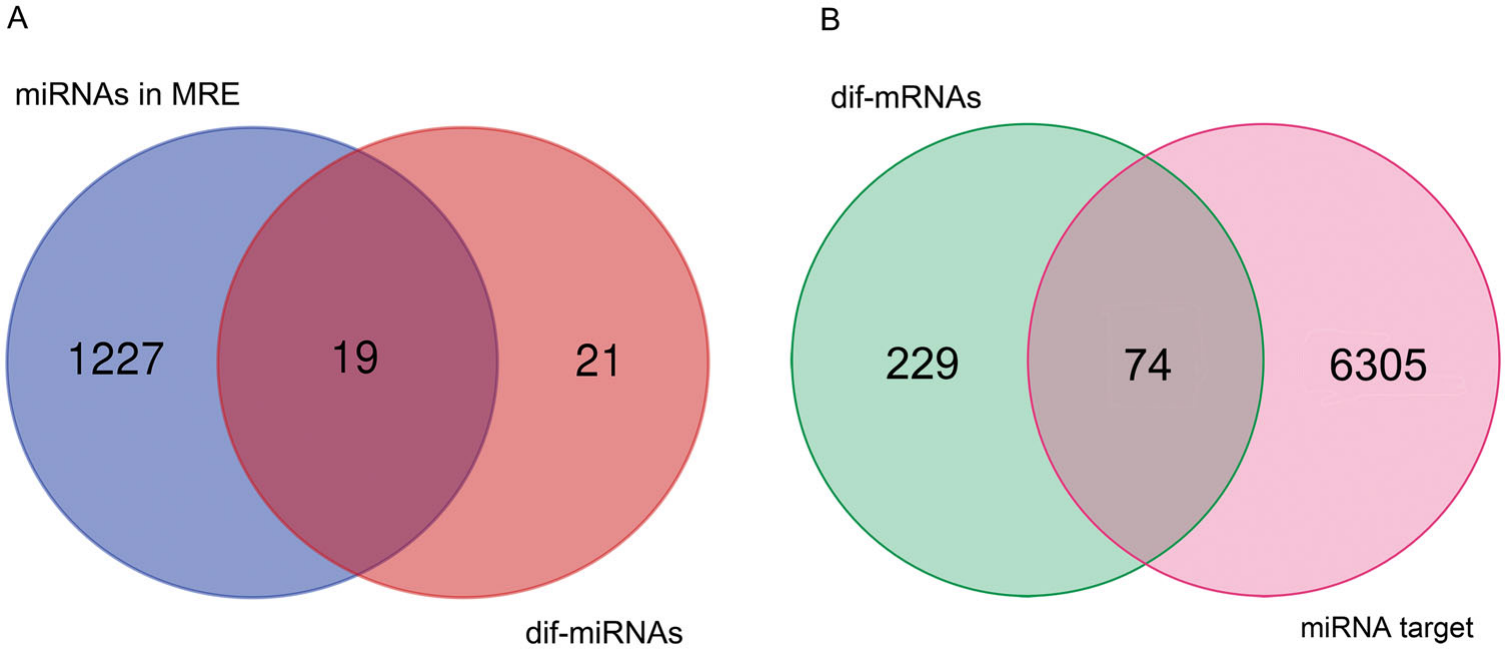
Venn results. (A) Venn result between dif-miRNAs and miRNAs in miRNA response elements (MRE). (B) Venn result between dif-mRNAs and mRNAs predicted as targets of miRNAs in the overlap in A.

A total of 1227 circRNA-miRNA interaction pairs were obtained from the circBank database by searching the dif-circRNAs, including 19 dif-miRNAs (Figure 5(A)). Then, 6305 miRNA–mRNA interaction pairs were obtained from two miRNA databases by using R, which contained 74 dif-mRNAs (Figure 5(B)).

### CircRNA–miRNA–mRNA regulatory network analysis

**Figure 6.**
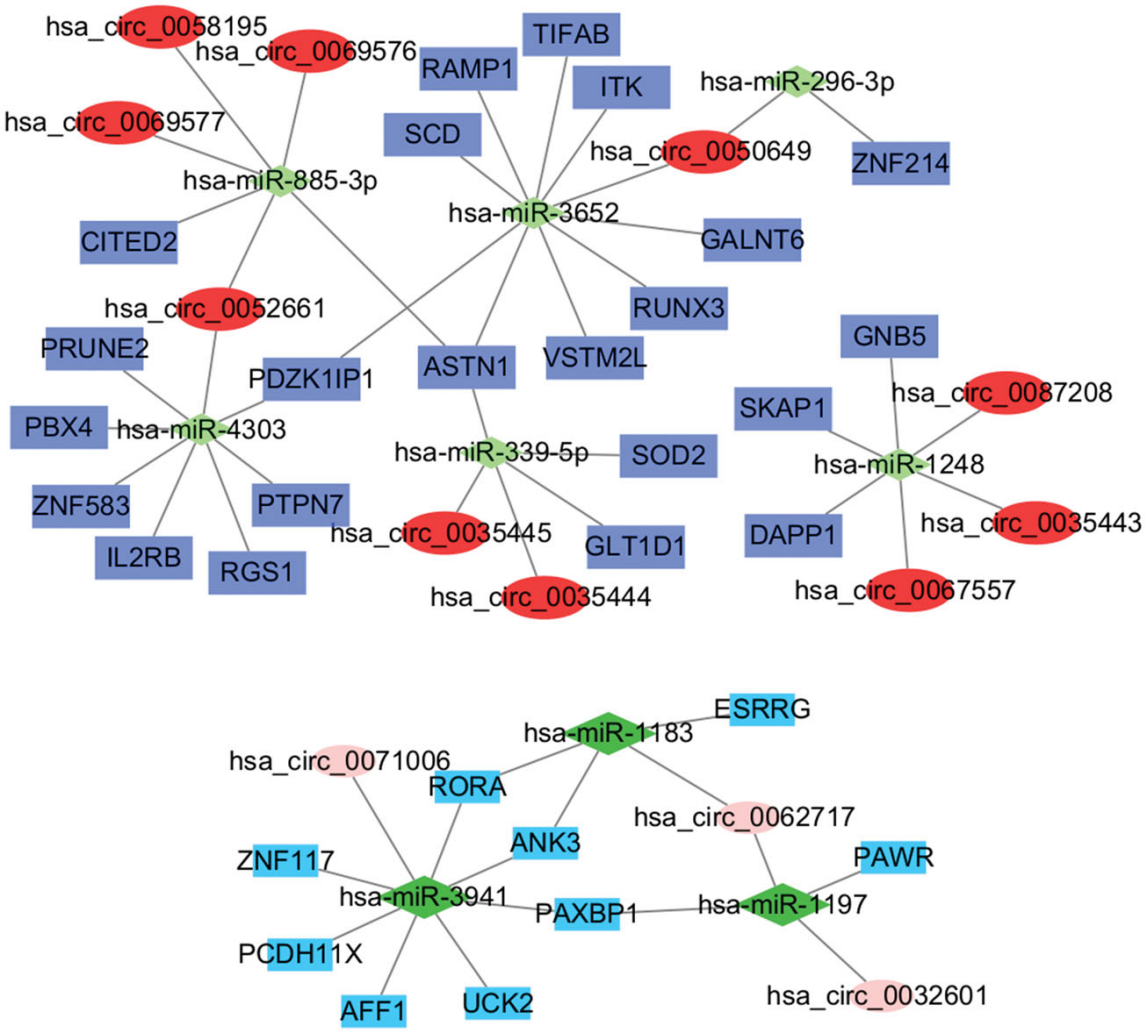
The circRNA-miRNA-mRNA regulatory network. The ellipses, diamonds and squares represent mRNAs, miRNAs and mRNAs, respectively. The dark colour means upregulated expression and light one means downregulation.

The data of 74 dif-miRNA-regulated mRNAs, 19 circRNA-related-dif-miRNAs and dif-circRNAs were analyzed by Perl to obtain the circRNA–miRNA–mRNA pairs. Fifty-three pairs were obtained, including 13 circRNAs (10 upregulated and 3 downregulated), 9 miRNAs (3 upregulated and 6 downregulated) and 31 mRNAs (22 upregulated and 9 downregulated) (Figure 6).

**Figure 7.**
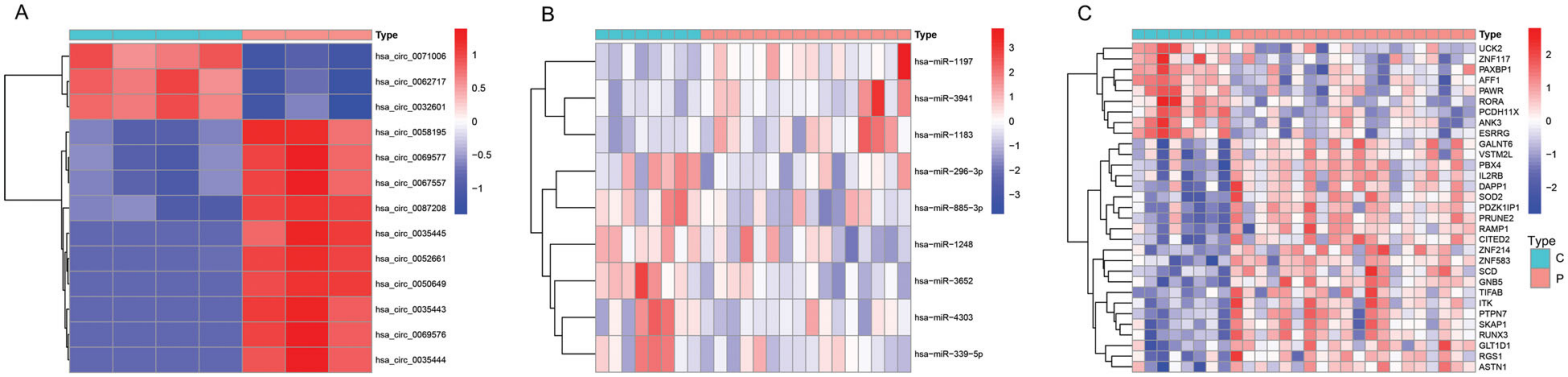
The bidirectional clustering heat maps of circRNAs (A), miRNAs (B), and mRNAs (C) in the circRNA–miRNA–mRNA regulatory network. C: control group; P: PE group; Dif-circRNAs/miRNAs/mRNAs are shown in different shades.

**Figure 8.**
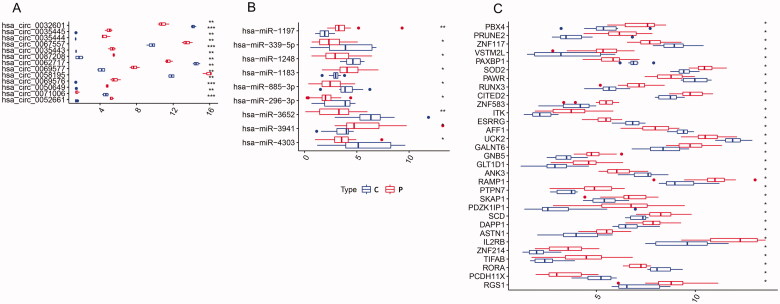
Boxplot of circRNAs (A), miRNAs (B), and mRNAs (C) in the circRNA–miRNA–mRNA regulatory network. C: control group; P: PE group; Up- and downexpressed circRNAs/miRNAs/mRNAs are shown in different shades. Asterisks represent Adj-*p*/*p*-value. *** means *p*<.001. ** means *p*<.01. * means *p*<.05.

**Figure 9.**
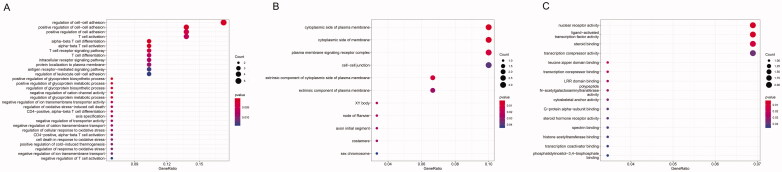
Functional enrichment of mRNAs in the circRNA–miRNA–mRNA regulatory network. The mRNAs in ceRNA are primarily involved in regulation of cell–cell adhesion in BP (A), cytoplasmic side of plasma membrane in CC (B), and nuclear receptor activity in MF (C).

Differential expression of these circRNAs, miRNAs and mRNAs between PE and control tissues is shown in Figures 7 and 8. Functional enrichment analysis of the 31 mRNAs in the ceRNA network is displayed in Figure 9. After taking the intersection between the mRNAs in the ceRNA network and in dif-mRNA modules by Venn diagram, we obtained two key genes: G Protein Subunit Beta 5 (GNB5) and Interleukin 2 Receptor Subunit Beta (IL2RB). Previous KEGG enrichment analysis showed that both of these genes were major components in the PI3K-Akt signalling pathway. Therefore, hsa_circ_0087208, hsa_circ_0035443 and hsa_circ_0067557 might function as ceRNAs affecting the PI3K-Akt signalling pathway by upregulating GNB5 by binding to miR-1248 in PE. Meanwhile, hsa_circ_0052661 might upregulate IL2RB by binding miR-4303 to take part in PE *via* the same mechanism.

## Discussion

PE is characterized by a variety of aetiologies and pathogenesis, a long subclinical period, clinical manifestations caused by affection on maternal multiple organ functions, foetal involvement, and the interactions of genetics and environment [24]. It can occur during the antepartum and postpartum periods [25]. We focus on antepartum here, because the tissues of PE were all from the former. Risk factors include maternal comorbidities, such as chronic kidney disease, hypertension and obesity; a family history of PE, nulliparity or multiple pregnancies; and previous PE or intrauterine foetal growth restriction [26]. Due to its possible serious conditions, including eclampsia, HELLP syndrome, placental abruption, and even multiorgan dysfunction [27], adequate and proper prenatal care is the most important part of the management of PE [28]. However, in the early stage, many patients with a pregnancy-induced hypertensive disorder have no clinical symptoms. Therefore, PE can only be reliably detected in the second half of pregnancy [29], when the symptoms appear, which also means that harm to the mother and child has already happened. New targets for the prediction and earlier diagnosis and treatment of PE need to be researched.

In this study, a total of 163 dif-circRNAs (61 upregulated and 102 downregulated), 39 dif-miRNAs (22 upregulated and 17 downregulated), and 271 dif-mRNAs (168 upregulated and 103 downregulated) between PE and control samples were obtained from 3 different datasets in the GEO database. GNB5 and IL2RB were identified as the key genes in the ceRNA network in PE by both PPI and circRNA-miRNA-mRNA networks. MiR-1248 targeted GNB5, and miR-4303 targeted IL2RB. Hsa_circ_0087208, hsa_circ_0035443 and hsa_circ_0067557 with miR-1248 and hsa_circ_0052661 with miR-4303 were predicted as ceRNAs affecting key genes that play roles in PE through the PI3K-Akt signalling pathway.

GNB5 encodes a beta subunit of heterotrimeric guanine nucleotide-binding proteins (G proteins), which is an important regulator of alpha subunits, as well as of certain signal transduction receptors and effectors. [provided by RefSeq, Jul 2008] GNB5 is closely involved in the PI3K-Akt signalling and other pathways in placental ischaemia, and it also took part in placental insufficiency and IUGR, which is consistent with the previous research results [10]. More researches on the direct correlation between GNB5 and PE should be conducted. Furthermore, GNB5 is involved in dopamine responses seen in neurons, and mutations in the gene are associated with a neuropsychiatric disorder that affects cognition [30]. It is recognized that PE, especially severe preeclampsia, might cause neuropsychiatric disorders in both mothers and children [31–33]. Therefore, based on our results, it is highly possible that GNB5 plays an important role in PE, at least as a key gene in a ceRNA network, which is worth further study.

The protein encoded by the IL2RB gene represents the beta subunit (IL2Rβ) and is a type I membrane protein that is primarily expressed in the haematopoietic system. It also can be specifically expressed in placenta induced by some variants of an alternate promoter in an upstream long terminal repeat (LTR). [provided by RefSeq, Sep 2016] In placenta tissue, IL2RB is expressed primarily in trophoblast cells from the LTR promoter and that its expression is regulated by DNA methylation, DNA methylation levels were inversely correlated with expression level [34]. IL2RB has been reported to be closely related to PE and might even contribute to the development of the early form of severe preeclampsia, as one of retrotransposons function as alternative promoters for placental-specific transcripts [35]. The IL-2Rβ chain is a key component of a functional cytokine receptor that responds to either interleukin-2 (IL-2) or interleukin-15 (IL-15), both of which were required at the maternal–foetal interface for differentiation and proliferation of decidual NK cells [36]. In a PE mouse model induced by lipopolysaccharide (LPS), IL-15 positively regulated IFN-γ expression. Inhibition of IL-15 decreased SBP and urine protein, alleviated kidney injury and improved pregnancy outcomes [37]. However, the direct function and underlying molecular mechanism of the trophoblast-specific transcript of IL2RB in PE is still ambiguous, which is also worth further exploration.

Both GNB5 and IL2RB were highly associated with the PI3K-Akt signalling pathway. The PI3K/AKT signalling pathway regulates a wide range of cellular functions, including cell growth, proliferation, migration and invasion [38]. Phosphatidylinositol 3-kinase (PI3K) activation phosphorylates and activates protein kinase B (PKB/AKT), localizing it in the plasma membrane. AKT can have a number of downstream effects, such as activating CREB, inhibiting p27, localizing FOXO in the cytoplasm, activating PtdIns-3ps, and activating mTOR, which can affect the transcription of p70 or retinol-binding protein 4 (4EBP1) [39]. It has been reported that many biological molecules influence the occurrence and development of PE *via* the PI3K/AKT signalling pathway [40]. As an inflammatory disease, PE can induce oxidative stress in the placenta *via* the PI3K/Akt/mTOR pathway [41]. Li et al. re-ported that RBP4 regulated proliferation and invasion of the trophoblastic cell *via* the PI3K/AKT signalling pathway. Decreased expression of RBP4 in the placenta may contribute to PE development by decreasing the invasive ability of trophoblasts [42]. Annexin IV (ANXA4) expression is downregulated in human placentas in PE. But in PE rats, overexpression of ANXA4 alleviated PE progression by promoting the invasion of trophoblast cell *via* activation of the PI3K/Akt/eNOS pathway [43]. Further investigation showed that exosome derived miR-15a-5p suppressed proliferation, invasion, and apoptosis of trophoblast cell by downregulating CDK1 to inhibit the activation of the PI3K/AKT pathway. In PE mice, placental exosomes treated with miR-15a-5p inhibitor attenuated histopathologic changes and apoptosis in the placenta. The exosomal miR-15a-5p could be a promising biomarker and therapeutic target in PE [44].

## Conclusions

We found that GNB5 and IL2RB were the key genes in the circRNA-miRNA-mRNA networks of PE. Hsa_circ_0087208, hsa_circ_0035443 and hsa_circ_0067557 with miR-1248 and hsa_circ_0052661 with miR-4303 were predicted as ceRNAs affecting GNB5 and IL2RB genes in the PI3K-Akt signalling pathway. This is a retrospective and *in silico* study that builds on the material presented in the GEO database. All three analyzed data sets were obtained by microarray technology, which is not the most sensitive tool to detect the low abundant RNAs. So the analysis is limited by the methodology. And not all 3 datasets used the same array platform, which might also influenced the obtained conclusions. Further experimental studies are needed to validate and demonstrate the definite functions of these key factors in PE. Even more advanced methods could be used to profile RNA expression, such as next-generation sequencing (NGS) technology, and Droplet Digital PCR (ddPCR) for the quantification of low abundant microRNAs. A strength of our study is that it provides a new research direction on a ceRNA network in PE. Based on this novel genetic interaction, we can further investigate the related molecular mechanism in PE pathophysiology and potential direction for new biomarker discovery.

## Supplementary Material

Supplemental Material

## Data Availability

The data that support the findings of this study are openly available in GEO at https://www.ncbi.nlm.nih.gov/geo/, Ref. [11].
