# Supplementary material for: Analysis of a circRNA-, miRNA-, and mRNA-associated ceRNA network reveals potential biomarkers in preeclampsia a ceRNA network in preeclampsia
Source: Ann Med. 2021 Dec 13;53(1):2354–64. doi: 10.1080/07853890.2021.2014554 (PMC8741177; doi:10.1080/07853890.2021.2014554)
Supplement: Supplemental Material [file IANN_A_2014554_SM7377.docx]

Table S1. Summarize of the preeclamptic and control participant characteristics we used from 3 datasets.

|  | GSE96984* | | GSE103542 | | | GSE147776 | | |
| --- | --- | --- | --- | --- | --- | --- | --- | --- |
|  | PE(n=3) | Controls(n=4) | EOPE^1^(n=11) | LOPE^2^(n=5) | Controls(n=8) | PE(n=7) | PE-IUGR^3^(n=6) | Controls (n=8) |
| Maternal Age (Years) | 29.33[26-32] | 30.25[28-33] | 35.1[28-45] | 28.4[20-35] | 35.7[35-39] | 30.57±1.5 | 30.33±2.6 | 30.57±6.18 |
| Pre-pregnancy BMI^4^(kg/m^2^) | Unavailable | Unavailable | 29.9[19.83-41.77] | 28.6[24.02 -36.57] | 29.2[22.96-31.99] | 29.9±1.4 | 25.36±1.66 | 24.3±0.9 |
| SBP^5^(mmHg) | Unavailable | Unavailable | 197.75[160-250] | 172.25[152-197] | 121[122-118] | 167.8±3.1 | 171.6±3.8 | 103.5±3.8 |
| DBP^6^(mmHg) | Unavailable | Unavailable | 113.5[90-150] | 107.75[94-135] | 75[72-85] | 112.14±2.85 | 116.6±1.9 | 68±1.75 |
| Gestational Age at birth(Weeks) | Unavailable | Unavailable | 31.2[28-33.5] | 35.6[34.4-37.3] | 36.3[34.2-38.5] | 32.9±0.98 | 32.01±1.15 | 38.5±0.48 |
| Delivery Type | Cesarean Section | | | | | | | |
| Newborn weight (g) | Unavailable | Unavailable | 1335[1025-1540] | 2454[1900-3020] | 2792.6[2215-3560] | 1958±176.79 | 1136.66±176.79 | 3167±130.69 |

*The paper based on GSE96984 has not yet been published, so many characteristics details are still unavailable.

1 EOPE: Early onset Preeclampsia

2 LOPE: Late onset Preeclampsia

3 IUGR: Intrauterine growth restriction

4 BMI: Body Mass Index

5 SBP: Systolic Blood Pressure

6 DBP: Diastolic Blood Pressure
